# Longitudinal Progression of Essential Tremor: Do Tremor Severity Scores Increase at a Uniform Rate?

**DOI:** 10.3389/fneur.2022.871905

**Published:** 2022-05-31

**Authors:** Margaret M. McGurn, Diane S. Berry, Jordan D. Dworkin, Elan D. Louis

**Affiliations:** ^1^Department of Neurology, University of Texas Southwestern Medical Center, Dallas, TX, United States; ^2^Department of Psychiatry, Vagelos College of Physicians and Surgeons, Columbia University, New York, NY, United States; ^3^New York State Psychiatric Institute, New York, NY, United States

**Keywords:** essential tremor, clinical, longitudinal, progression, Archimedes spiral

## Abstract

**Background:**

Few longitudinal studies assess the progression of essential tremor (ET). One unexplored issue is whether tremor severity increases across time at a uniform rate. That is, does the observed rate of change in tremor severity within a particular patient remain constant or vary across time? This question of intra-individual differences is particularly important since it reflects a primary patient concern–will the nature of change I have seen to date be what I can expect in the future?

**Methods:**

ET cases were enrolled in a prospective, longitudinal study. We selected 35 cases and assessed tremor severity via Bain and Findley ratings of Archimedes spirals assigned by a senior movement disorders neurologist. After reviewing both the change in spiral scores and the rate of change in scores, we identified five mutually exclusive patterns of severity change. We calculated the prevalence of each category using two complementary sets of classification criteria.

**Results:**

Length of follow-up was 4.5 to 16.0 years, mean=10.2 years. Mean baseline tremor severity score was 4.6, SD=1.6. Depending upon the classification criteria used, the tremor scores of one-third to one-half of cases did not increase in a uniform fashion but were better described as demonstrating jumps and/or reversals in scores across time.

**Conclusions:**

We document the nature of changes in ET tremor severity scores across a ten-year period via expert ratings of Archimedes spiral drawings. Such natural history data are valuable to patients and clinicians who hope to better understand and predict the likely course of ET symptoms.

## Introduction

Essential tremor (ET) is a highly prevalent neurological disease (1). Although generally regarded as progressive in nature, the course of this disease is not well understood as few longitudinal clinical studies of ET patients are available (2). The best-documented aspect of ET progression is the average rate at which tremor severity increases. For example, analyses of data from two ET cohorts yielded average and median annual increases in tremor severity of 3.1–5.3% and 1.8–2.0% above baseline, respectively, over 4.3 to 5.7 year average follow-up intervals (3). Similarly, an analysis of tremor progression rates derived from an 11-point spiral rating scale yielded an average annual increase in tremor severity of 0.12 ± 0.23 points over a mean follow-up interval of 5.8 years (4).

A related issue involves *variability* in the progression of tremor severity. First, does the observed rate of change in tremor severity within a particular patient remain constant or vary across time? This question of intra-individual differences is particularly important because it reflects a primary patient concern in clinical settings–will the nature of change I have seen to date be what I can expect in the future? If there is within-patient variability in tremor severity change, a second question arises: is the nature of this variability the same for all patients, or do different patterns of change emerge across time for different people?

Our initial goal was to determine whether tremor severity scores in ET cases typically increase at a uniform rate across time, or whether there are intra-individual differences in tremor severity progression. Our recent observations suggest that such intra-individual differences may characterize tremor progression for at least some ET cases. For example, we compiled videos of three ET patients performing a series of movement tasks at subsequent observation points. Analyses of these videos suggest that the progression of tremor ratings in ET, at least for some patients, is best described as non-uniform, or irregular (5) (e.g., years of mild tremor followed by a sudden, pronounced increase in tremor severity). In the current analysis, we documented the occurrence of such intra-individual differences in tremor severity scores in the ET patient population, and subsequently identified different patterns of within-patient change across time. Finally, we examined whether individual differences, such as sex, age of tremor onset, or changes in medication use or alcohol consumption predict these particular patterns of change.

## Methods

### Cases

Participants were enrolled as future brain donors in the Essential Tremor Centralized Brain Repository (ETCBR, 2003 - present), a prospective, longitudinal clinical-pathological study of ET. In addition to the measures we report, this cohort has provided a wealth of clinical and neuropsychological data described in detail elsewhere (4, 6, 7). The University of Texas Southwestern Medical Center, Yale University, and Columbia University Institutional Review Boards approved study procedures, and all participants signed informed consents.

In this longitudinal study, evaluations were performed on multiple, sequential occasions. Specifically, the 35 cases in this sample were observed for a mean of eight time intervals, with a mean length of follow-up of 10.2 ± 3.2 years. At baseline, participants completed clinical questionnaires to collect demographics and information relevant to ET. They also completed a shortened version of a cognitive screening test (the Telephone Interview for Cognitive Status, TICS, range = 0 [most impaired] – 9) (8). Data on age of tremor onset, average weekly alcohol consumption, and tremor medications were collected as well.

Participants also completed a videotaped neurological examination that included tests of postural, kinetic, rest, and intention tremor, as well as tremors in the head, voice, and other body regions. One of the videotaped kinetic tremor tests involved drawing a set of four standardized Archimedes spirals (two by the dominant arm, two by the non-dominant arm) on 8.5” by 11” sheets of paper (6). While seated at a table, participants freely drew these spirals with a ballpoint pen. They started in the center of the paper and did not lift the pen at any point in the drawing. The paper was oriented vertically (i.e., portrait orientation). Participants could secure the paper with their non-drawing hand, but we required that the drawing hand and arm remain unsupported (i.e., not resting on the table). Upon completion, a senior movement disorders neurologist (EDL) (6), reviewed the videotaped examinations, rated tremor severity, and confirmed ET diagnoses using reliable and valid diagnostic criteria (9–11).

Participants who agreed to follow-up were asked to complete the aforementioned clinical questionnaires over the phone and provide spiral drawings—produced following the identical procedure previously described—every six to nine months from 2009 to 2013 (4). Some participants agreed to further follow-up by enrolling in the Clinical Pathological Study of Cognitive Impairment in Essential Tremor (COGNET), a longitudinal study that began in 2014 (7). Follow-up in this study involved the aforementioned clinical questionnaires and videotaped neurological examination as well as comprehensive neuropsychological testing. Participants in this study completed the evaluation at regular 18-month intervals.

### Final Sample

Our analysis focuses on the spirals drawn during the aforementioned videotaped examination or follow-up clinical assessments. We included data from all cases who (1) had a confirmed diagnosis of ET with no additional diagnoses at baseline (e.g., dystonia); (2) did not have brain surgery for treatment of tremor at any point during follow-up; and (3) completed the required study evaluations. This yielded a sample of 141. We further excluded participants who (1) completed fewer than four study evaluations (*n* = 28); (2) did not complete a minimum of 4.5 years of follow-up (*n* = 22); or (3) provided spirals that received the maximum possible tremor rating score (i.e., were at ceiling) at the time of enrollment (*n* = 56), yielding a final sample of 35 (54.3% men, mean age at baseline = 76.9 years, Table 1), who completed 267 study evaluations involving spiral drawings. One-hundred and twenty of these spiral drawings were completed during the videotaped neurological examination, and the remaining 147 were spiral drawings completed during the abbreviated version of follow-up. In sum, we evaluated 1,068 spirals (i.e., 267 × 4 spirals per study evaluation).

**Table 1:** Baseline characteristics of ET cases (*n* = 35).

**Characteristic**	**Mean ±SD / frequency (percentage)**	**Median**	**Range**
Age (years)	76.9 ± 8.65	78.6	45.3–90.3
Sex (male)	19 (54.3)	–	–
Right-handed	30 (85.7)	–	–
Family history of tremor (yes)	28 (80.0)	–	–
Tremor onset age (years)	39.4 ± 24.6	40.0	5.0–82.0
Tremor duration (years)	37.5 ± 21.8	32.6	5.6–75.2
Number of spiral sets	7.8 ± 2.1	8.0	4.0–11.0
Period of follow-up (years)	10.2 ± 3.2	9.7	4.5–16.0
Total arm tremor score	4.58 ± 1.57	–	–
Surgery for tremor	0(0.0)	–	–
Shortened TICS score	8.88 ± 0.49	9.0	7.0–9.0

*TICS, telephone interview for cognitive status*.

### Spiral Ratings

EDL rated the tremor severity of each hand-drawn paper spiral using the ordinal clinical rating scale developed by Bain and Findley (12). This 11-point scale (0 = no detectable tremor; 10 = severe tremor) has well-established validity and reliability (12–15). In fact, pervious literature has established that inter-rater reliability ranges from 0.56−0.90 (moderate to almost perfect) (12). EDL reviewed randomly ordered spiral sets (using a randomly assigned number for each set), and rated all four spirals from the same set (i.e., participant X and study evaluation Y) together. The spiral sets of a given participant were randomly ordered with respect to time, and they were mixed with those of other participants. Thus, EDL did not see all the spiral sets of a given participant in the order they were drawn or all at once. Additionally, EDL was blinded to any clinical information of the participants.

We averaged the ratings of the two dominant arm spirals to yield a “dominant arm tremor score” and the ratings of the two non-dominant arm spirals to produce a “non-dominant arm tremor score.” The four ratings were further averaged to produce a “total arm tremor score.”

### Classification of Tremor Score Progression Patterns

We calculated the rate of change in the tremor score between each adjacent pair of study evaluations by dividing the difference between the two scores by the time in years elapsed between the two study evaluations. We computed these values separately for the total, dominant, and non-dominant arm tremor scores. To identify different patterns of progression, we plotted tremor scores for each of the 35 participants over time. An initial examination of the data revealed that none of the cases exhibited a pattern of no change (defined as receiving the same score at each study evaluation), or evidence of a ceiling effect (defined as a score equaling the maximum value at any study evaluation).

**Figure 1:**
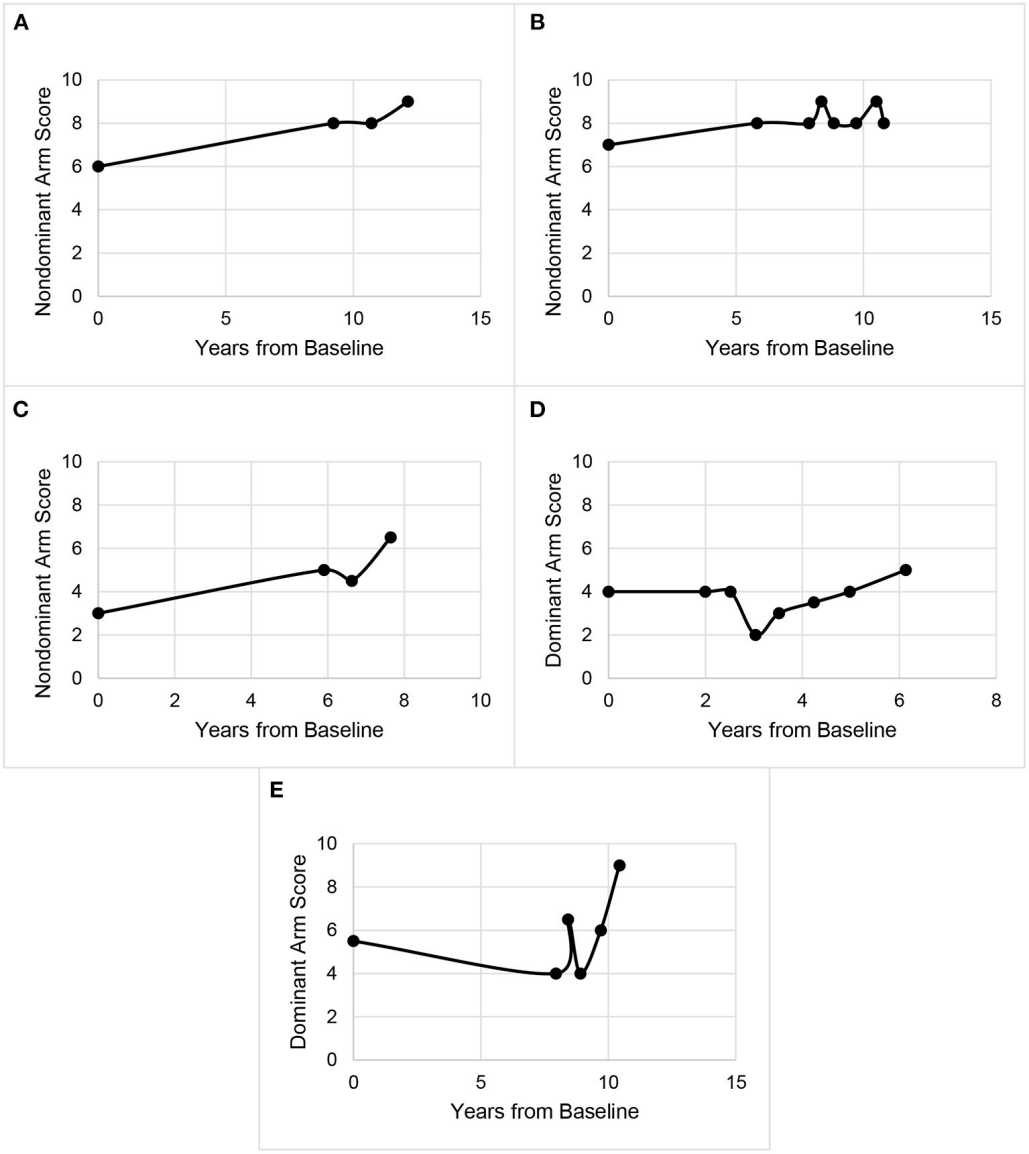
Example plots of tremor score progression patterns. **(A)** Overall increase, **(B)** small fluctuations, **(C)** jump occurs between year 6 and 8, **(D)** reversal occurs between years 2 and 4, and **(E)** jumps and a reversal.

We categorized the tremor progression of each participant into five mutually exclusive categories via a joint consideration of the change in tremor scores and the rate of change in tremor scores between consecutive evaluations. Categories were assigned independently for scores observed for the dominant arm, the non-dominant arm, and for both arms. Sample plots of each tremor progression category appear in Figure 1. The categories included ‘Overall Increases,’ ‘Small Fluctuations,’ ‘Jumps,’ ‘Reversals,’ and ‘Jumps and Reversals’ (Operational definitions appear in Table 2). We carefully defined the criteria to distinguish ‘Small Fluctuations’ from ‘Jumps’ and/or ‘Reversals’ based on prior literature. In particular, we based our criteria on a previous study of spirals drawn by an ET cohort over a 1-hour period under identical conditions (16). Specifically, those investigators assessed normal intra-individual variability in Bain and Findley scale ratings of the spirals (i.e., background noise). As 95.4% of the absolute differences between spiral scores was <2, the authors concluded that changes <2 points could be attributed to normal intra-individual variability that represents background noise (16). Thus, we defined a notable change in tremor scores (i.e. a ‘Jump’ or ‘Reversal’) as occurring only if the shift was ≥2 points in magnitude. Any change smaller than two points was considered a ‘Small Fluctuation.’

**Table 2:** Tremor progression patterns.

**Tremor progression** **pattern**	**Scheme 1 definition**	**Scheme 2 definition**
Overall increase	The difference between the initial and final tremor scores was ≥ 2 points. No reversals or jumps were observed at any interval.	The difference between the initial and final tremor scores was ≥ 2 points. No reversals or jumps were observed at any interval.
Small fluctuations	For at least one-half of evaluations, the difference between the maximum and minimum tremor score was <2 points. Additionally, the difference between the initial and final scores was <2 points. No reversals or jumps were observed at any interval.	For at least one-half of evaluations, the difference between the maximum and minimum tremor score was <2 points. Additionally, the difference between the initial and final scores was <2 points. No reversals or jumps were observed at any interval.
Jumps	At one or more evaluations, the tremor score increased by at least 2 points, with a rate of change ≥1 points/year	At one or more evaluations, the tremor score increased by at least 2 points, with a rate of change ≥2 points/year
Reversals	At one or more evaluations, the tremor score decreased by at least 2 points, with a rate of change ≤ -1 points/year.	At one or more evaluations, the tremor score decreased by at least 2 points, with a rate of change ≤ -2 points/year.
Jumps and reversals	At one or more evaluations, there was a reversal. Additionally, at one or more evaluations, there was a jump.	At one or more evaluations, there was a reversal. Additionally, at one or more evaluations, there was a jump.

We also considered the rate of change in the tremor score when defining ‘Jumps’ and/or ‘Reversals.’ Consider two hypothetical ET patients. Patient 1 experiences a 2-point change in his tremor score over 4 years. Patient 1's rate of change is 0.5 points per year (two points divided by 4 years). Meanwhile, Patient 2 experiences the same 2-point increase, but over 2 years, yielding a rate of change of 1 point per year. Although these patients experience the same amount of change, Patient 1's progression is more gradual than that experienced by Patient 2. Thus, the rate of change in tremor scores, in addition to the magnitude of change, was an important factor in our definition of ‘Jumps’ and/or ‘Reversals.’ Although there is no established guideline for a significant rate of change in tremor scores, a study of spirals drawn by 116 ET cases and rated with the same Bain and Findley scale found an average rate of 0.12 ± 0.23 points and a maximum rate of 1 point per year (4). Therefore, we decided that a rate greater than or equal in magnitude to 1 point per year likely does not reflect gradual changes in tremor severity and is sufficient for defining ‘Jumps’ and/or ‘Reversals.’

In sum, we based these operational definitions of jumps and reversals (Scheme 1; Table 2) on previously reported data. However, we acknowledge that the definition of what constitutes a ‘true’ jump or reversal has yet to be formally established. Thus, to enhance methodological rigor, we developed a second set of criteria (Scheme 2; Table 2) that differed from Scheme 1 only in the stringency of its inclusion rules for the categories of ‘Jumps’, ‘Reversals’, and ‘Jumps and Reversals.’ Whereas Scheme 1 based its cutoffs directly on previous research data (4, 16), Scheme 2 adopted more stringent criteria. Specifically, we raised the required rate of change in tremor scores for ‘Jumps’ to +2 points per year and for ‘Reversals’ to −2 points per year. Consequently, Scheme 2 requires qualifying participants to experience a minimum rate of change *double* the rate of 1 point per year observed in the aforementioned study (4). Thus, the implementation of Scheme 2 even further minimizes the likelihood that any changes identified as jumps and reversals reflect normal fluctuations (i.e., normal background noise).

### Predictors of Tremor Progression Patterns

We examined the ability of several variables to predict patterns of tremor progression. These included sex, age of tremor onset, disease duration, changes in tremor medication, and changes in alcohol consumption. Information about gender, age of onset and disease duration were derived from clinical questionnaires administered at baseline.

We collected self-reports of medication use at each time interval. Specifically, all cases reported their use of propranolol and/or primidone, the two frontline medications for ET. We classified all cases as either reporting no change or a change in the use of these medications (e.g., discontinuing or adding either medication) from each interval to the next. Information about propranolol and/or primidone dosage (mgs/day) was further provided by cases who participated in COGNET (*n* = 13). We used these data to classify cases as either changing or not changing their daily medication dosage at each time interval.

Information about alcohol consumption was also collected at each time interval. Specifically, cases reported the average number of alcoholic drinks consumed weekly. We classified cases as reporting either a change or no change in weekly alcohol consumption at each point in time. “Change” was operationally defined as an increase or decrease in the weekly reported average alcohol intake of at least one beverage from one time interval to the next.

### Head and Voice Tremor

Although our focus was on tremor progression in the upper limbs, we had access to some longitudinal data on head and voice tremors. Specifically, the presence versus absence of head and voice tremors was ascertained via expert ratings of cases' videotaped neurological examinations conducted at baseline and at subsequent evaluations during the COGNET study (*n* = 26, 105 evaluations). Thus, we determined the frequency of cases whose head and/or voice tremor was present at baseline or emerged at a follow-up evaluation. We then assessed (yes/no) whether or not the presence of head and/or voice tremor was stable over time—that is, present at every evaluation following its inception. For these cases whose head and/or voice tremor was not stable over time, we described the pattern of instability: did it disappear at one evaluation and re-emerge at a subsequent one or did it disappear completely? Finally, when head and/or voice tremor emerged or disappeared from one evaluation to the next, we determined whether this change co-occurred with a ‘Jump’ or ‘Reversal’ in the total arm tremor score.

### Statistical Analysis

The proportion of cases assigned to each tremor progression category were calculated based on the criteria described in Scheme 1, and again using the criteria described in Scheme 2. Chi-square tests assessed whether proportions differed as a function of pattern and classification scheme. We further employed chi-squares and Kruskal-Wallis tests to assess whether potential predictor variables differed as a function of tremor progression patterns.

## Results

### Frequency of Tremor Progression Patterns

The proportion of participant tremor progression patterns ascribed to each category for the total arm tremor scores did not differ as a function of the set of inclusion rules employed, *X*^2^(4)= 3.62, *p* = 0.46 (Table 3a, b). Collapsing across the two sets of categorization rules, ‘Overall Increase’ was most frequently observed (35.7%), followed by ‘Reversals and Jumps’ (22.9%), ‘Small Fluctuations’ (21.4%), ‘Jumps’ (12.9%), and ‘Reversals’ (7.9%). Although ‘Overall Increase’ was indeed the most frequently observed pattern of tremor progression, ‘Reversals’ and/or ‘Jumps’ together described the patterns experienced by one third to one half of patients, depending on the classification scheme employed. See Supplemental Figures 1a–d, showing individual-level data, stratified by age of onset for ease of viewing.

**Table 3A:** Frequency of tremor progression patterns following scheme 1 definitions.

**Pattern**	**Total arm tremor score**	**Dominant arm tremor** **score**	**Non-dominant arm tremor score**
Overall increase	10 (28.6)	8 (22.9)	9 (25.7)
Small fluctuations	7 (20.0)	2 (5.71)	3 (8.57)
Jumps	5 (14.3)	10 (28.6)	7 (20.0)
Reversals	2 (5.7)	1 (2.9)	2 (5.7)
Jumps and reversals	11 (31.4)	14 (40.0)	14 (40.0)

*Frequency (percentage)*.

**Table 3B:** Frequency of tremor progression patterns following scheme 2 definitions.

**Pattern**	**Total arm tremor score**	**Dominant arm tremor** **score**	**Non-dominant arm tremor score**
Overall increase	15 (42.9)	15 (42.9)	13 (37.1)
Small fluctuations	8 (22.9)	5 (14.3)	6 (17.1)
Jumps	4 (11.4)	6 (17.1)	5 (14.3)
Reversals	3 (8.6)	2 (5.7)	2 (5.7)
Jumps and reversals	5 (14.3)	7 (20.0)	9 (14.3)

*Frequency (percentage)*.

### Predictors of Tremor Progression Patterns

We first examined the distribution of sex as a function of tremor progression pattern. Chi-square analyses revealed that women were more likely than men to display patterns involving ‘Jumps’ and/or ‘Reversals’ (75.0 vs. 31.6%), whereas men were more likely than women to exhibit ‘Overall Increases’ or ‘Small Fluctuations’ (68.4 vs. 25.0%), *X*^2^(1) = 6.56, *p* = 0.01. Cases' age of tremor onset also differed as a function of tremor progression pattern, *H*(2) = 6.95, *p* = 0.03. Specifically, *post-hoc* tests revealed that age of onset was significantly higher for cases displaying ‘Small Fluctuations’ (55.0 ± 23.6), than for cases displaying ‘Overall Increases’ (23.6 ± 15.8), *p* < 0.005; cases displaying ‘Reversals’ and/or ‘Jumps’ did not differ in onset age from either group (40.1 ± *2*6.0), *p*'s = 0.39 and 0.06, respectively. Finally, disease duration did not differ as a function of tremor progression pattern, *H*(2)= 5.30, *p* = 0.07.

Analyses of propranolol and primidone use revealed 21 changes in tremor medications across 15 participants. We further examined whether these changes co-occurred with either ‘Reversals’ or ‘Jumps’ in the total arm tremor score. The great majority of medication changes (18, 85.7%) were not accompanied by a reversal or jump in tremor scores, and analyses revealed no overall association between the presence versus absence of a change in medications and cases' tremor progression pattern, *X*^2^(2) = 1.90, *p* = 0.40. Examination of the available dosage data revealed that only six of 13 participants changed medication dosage. Of these six changes, the majority (5, 83.3%) did not co-occur with a ‘Reversal’ or ‘Jump’ in the total arm tremor score. Again, no overall association was revealed between the presence versus absence of a change in cases' medication dosage and their tremor progression pattern, *X*^2^(2) = 0.66, *p* = 0.72.

Analyses of alcohol use revealed 40 changes in weekly average alcohol consumption across 20 participants. The great majority of these changes (36, 90.0%) did not co-occur with a ‘Jump’ and/or ‘Reversal’ in the total arm tremor score. Moreover, chi-square statistics revealed no significant association between changes in alcohol consumption and the tremor progression patterns, *X*^2^(2) = 2.44, *p* = 0.30.

### Consideration of Tremors in the Head and Voice

Across 26 participants, there were 105 evaluations available for this analysis. Of these 26 participants, 21 evidenced head tremor (14 at baseline, seven during follow-up). In 15 of 21 participants, head tremor remained present at every interval following its inception. In total, there were seven changes in the presence of head tremor from one interval to the next, two of which were the emergence of head tremor and five of which were the disappearance of head tremor. One emergence and one disappearance co-occurred with a ‘Jump.’ The remaining five changes (71.4%) in the presence of head tremor did not co-occur with a ‘Jump’ or ‘Reversal.’

Regarding voice tremor, 20 participants had voice tremor (eight at baseline, 12 during follow-up). For 15 participants, voice tremor was present at every interval following its inception. In total, there were 11 changes in the presence of voice tremor from one evaluation to the next, seven of which were the emergence of voice tremor and four of which were the disappearance. Only two changes, the emergence of voice tremor, co-occurred with a ‘Jump.’ The remaining nine changes (81.8%) did not co-occur with a ‘Jump’ or ‘Reversal.’

## Discussion

A number of studies describe the average change in tremor ratings or change in accelerometric measures of tremor in ET patients over limited time periods (e.g., 4 years) (3, 4, 17, 18). To our knowledge, no published research documents either intra-individual differences in the progression of tremor severity, or the prevalence of specific patterns of tremor severity progression. We addressed these gaps in the literature by following the tremor severity displayed in a sample of ET cases across an average of 10.2 years and eight observations. Our assessments specifically focused on drawings of Archimedes spirals evaluated by a senior movement disorders neurologist using a standardized tremor severity scale.

A sizable proportion of cases displayed ‘Jumps’ and/or ‘Reversals’ in tremor severity scores across time. In fact, depending upon the classification criteria used, the tremor severity scores of one-third to one-half of patients did not increase in a uniform fashion, but were better described as demonstrating ‘Jumps’ and/or ‘Reversals’ in scores across time. This suggests that for a substantial number of patients, tremor does not increase uniformly over time. In fact, the irregular progressions witnessed in the previously described video compilation of three ET cases may illustrate the experiences of many ET patients (5). From a clinical standpoint, it is important for patients to realize that their tremor severity scores do not necessarily change in a uniform, steady, and regular manner over time. The change over time is instead characterized by a number of potential irregularities; if one were to plot their tremor scores over time, one would not necessarily see a smooth line (Supplemental Figures 1a–d).

An important direction for future research is the identification of factors that predict the progression patterns cases experience. Although we found no evidence that changes in tremor medication or alcohol consumption were associated with patterns of tremor progression, our data do suggest that women may be more likely than men to display jumps and/or reversals in the progression of their tremor. To our knowledge, sex differences in ET tremor progression have not received attention in the literature and constitute an important avenue for future research.

In most participants, head tremor remained present at every interval following its inception. The same was true for voice tremor. This being said, in some this was not the case. Head and voice tremor can be quite subtle in ET, and head tremor can be quite intermittent. In some cases without stable head tremor, this could have accounted for disappearance of head tremor from one interval to the next.

We acknowledge several limitations to our study. First, patients associated with ET-specific organizations or self-referred as future brain donors may be especially likely to experience relatively severe symptoms (3). Therefore, studies of ET patients with milder disease may be of value as they may better reflect the experience of progression for the entire ET population. Second, although we were careful to distinguish “tremor ratings” from “tremor severity”–as our ratings are derived from an ordinal rather than a continuous measure–future researchers may wish to use accelerometry to replicate these findings. Third, the sample size, *n* = 35, although adequate for our purposes, was modest and a larger sample would provide more precise estimates of the true proportions of tremor progression patterns. Additionally, for many cases, information about the daily dosage of tremor medications used by our cases was not available, forcing us to rely on a less fine-tuned measure of use vs. non-use of propranolol and primidone to predict tremor progression patterns. Moreover, these analyses were based on a small subset of cases, limiting their power to detect relations among these variables. Finally, as noted in the Methods section, in many cases follow-up involved telephone-derived data and spirals rather than a videotaped neurological examination; hence, we were able in a subsample rather than the full sample to comment on progression of head and voice tremor.

Finally, one might argue that the changes that occur across time in the severity of our patients' tremors are indeed regular and our reports of deviations from uniformity merely reflect small variations in the assignment of ordinal tremor ratings. However, this seems unlikely. First, one individual assigned each rating, eliminating inter-rater differences as a source of such variations. Moreover, sizable proportions of the cases we studied evidenced reversals and/or jumps. In addition, we carefully developed our definitions of these events, based on previous research (4, 16) to exceed the magnitude of what would be expected from random variations (‘background noise’) within participants.

This longitudinal study possesses notable methodological strengths. Upon enrollment in the ETCBR, cases were carefully assigned an ET diagnosis by a senior movement disorders neurologist using reliable and valid criteria (9–11). Similarly, all tremor ratings were assigned by a senior movement disorders neurologist using a standardized, reliable, and valid measure. Finally, we followed cases for a longer time than is typical in studies of this type [i.e., 10.2 years on average versus 5.6 years (3), 5.8 years (4), and 3.6 years (15)].

In sum, we document the non-uniform progression of ET tremor severity scores over time via a longitudinal analysis of Archimedes spiral drawings. The majority of our cases demonstrated considerable intra-individual variability in tremor severity ratings, highlighting the unpredictability of the progression of tremor over time for many individuals. We hope these data will help clinicians provide more useful responses to patients' concerns about the prognosis of their disease.

## Data Availability Statement

The raw data supporting the conclusions of this article will be made available by the authors, without undue reservation.

## Ethics Statement

The studies involving human participation were reviewed and approved by the Institutional Review Board at Columbia University, Yale University, and University of Texas Southwestern Medical Center. The patients/participants provided their written informed consent to participate in this study.

## Author Contributions

EL contributed to the conception and design of the study. MM organized the database and wrote the first draft of the manuscript. DB and JD performed the statistical analysis. DB wrote sections of the manuscript. All authors contributed to the article and approved the submitted version.

## Funding

This study was funded by the National Institutes of Health (NINDS R01 NS086736). The authors declare that there are no additional disclosures to report for this article.

## Conflict of Interest

The authors declare that the research was conducted in the absence of any commercial or financial relationships that could be construed as a potential conflict of interest.

## Publisher's Note

All claims expressed in this article are solely those of the authors and do not necessarily represent those of their affiliated organizations, or those of the publisher, the editors and the reviewers. Any product that may be evaluated in this article, or claim that may be made by its manufacturer, is not guaranteed or endorsed by the publisher.

## Supplementary Material

Supplemental Figures 1a–d Individual level data on tremor progression pattern. **(a)** Ages 0–20. **(b)** Ages 21–40. **(c)** Ages 41–60. **(d)** Ages 61 and above.
