# Supplementary figures and images for: Longitudinal Progression of Essential Tremor: Do Tremor Severity Scores Increase at a Uniform Rate?
Source: Front Neurol. 2022 May 31;13:871905. doi: 10.3389/fneur.2022.871905 (PMC9197457; doi:10.3389/fneur.2022.871905)

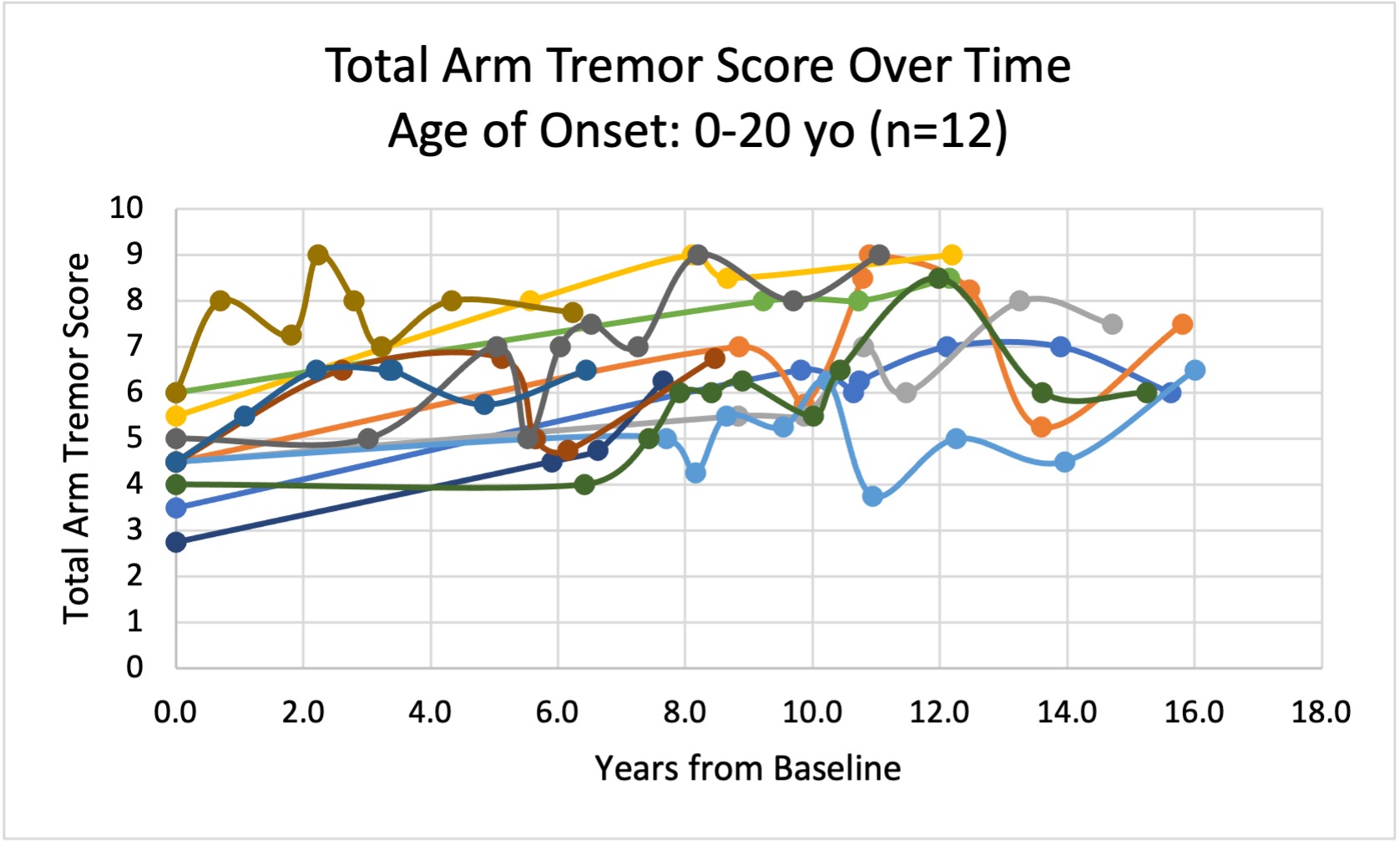

Supplement: Supplemental Figures 1a–d — Individual level data on tremor progression pattern. (a) Ages 0–20. (b) Ages 21–40. (c) Ages 41–60. (d) Ages 61 and above. [file Image_1.JPEG]

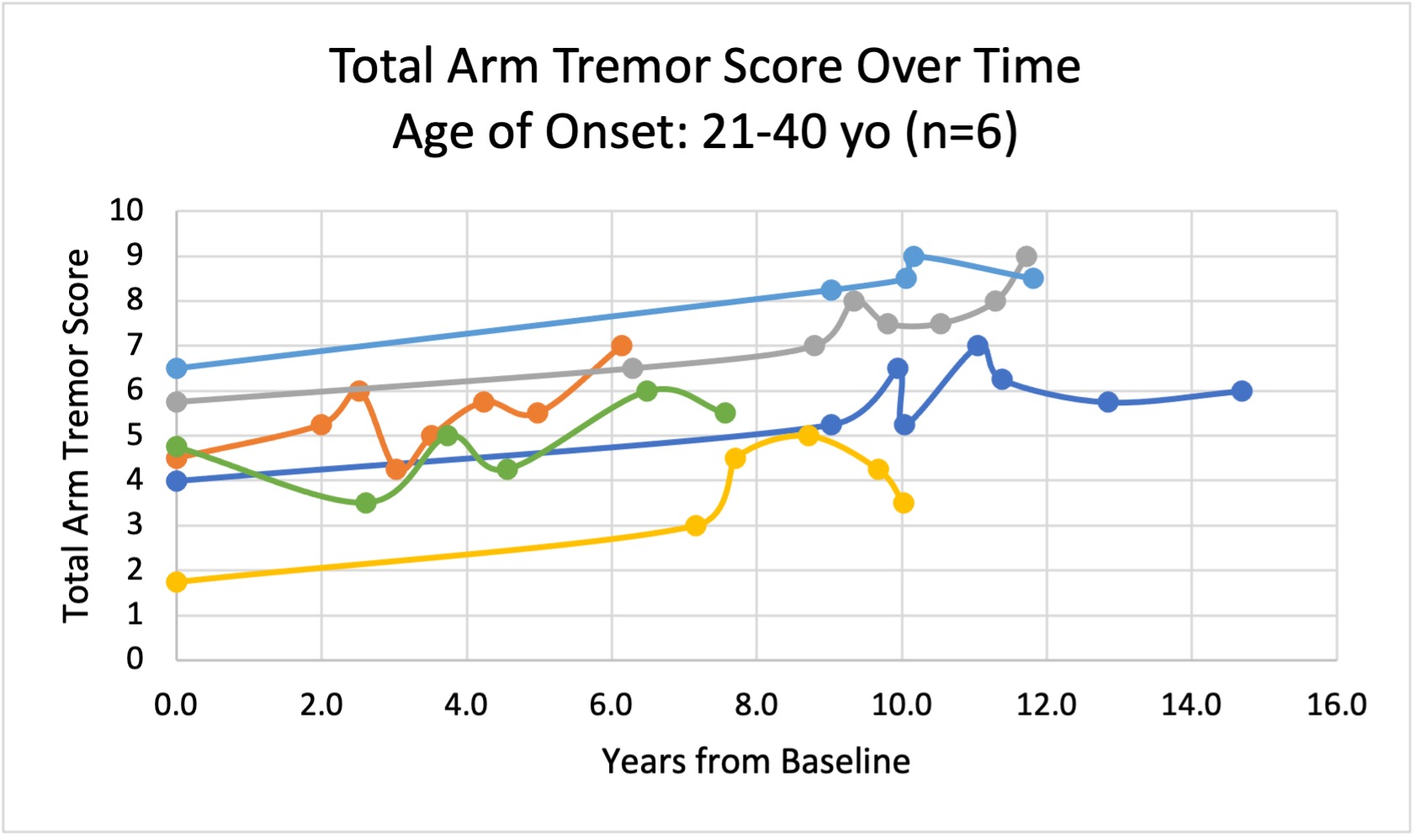

Supplement: Supplementary file 2 [file Image_2.JPEG]

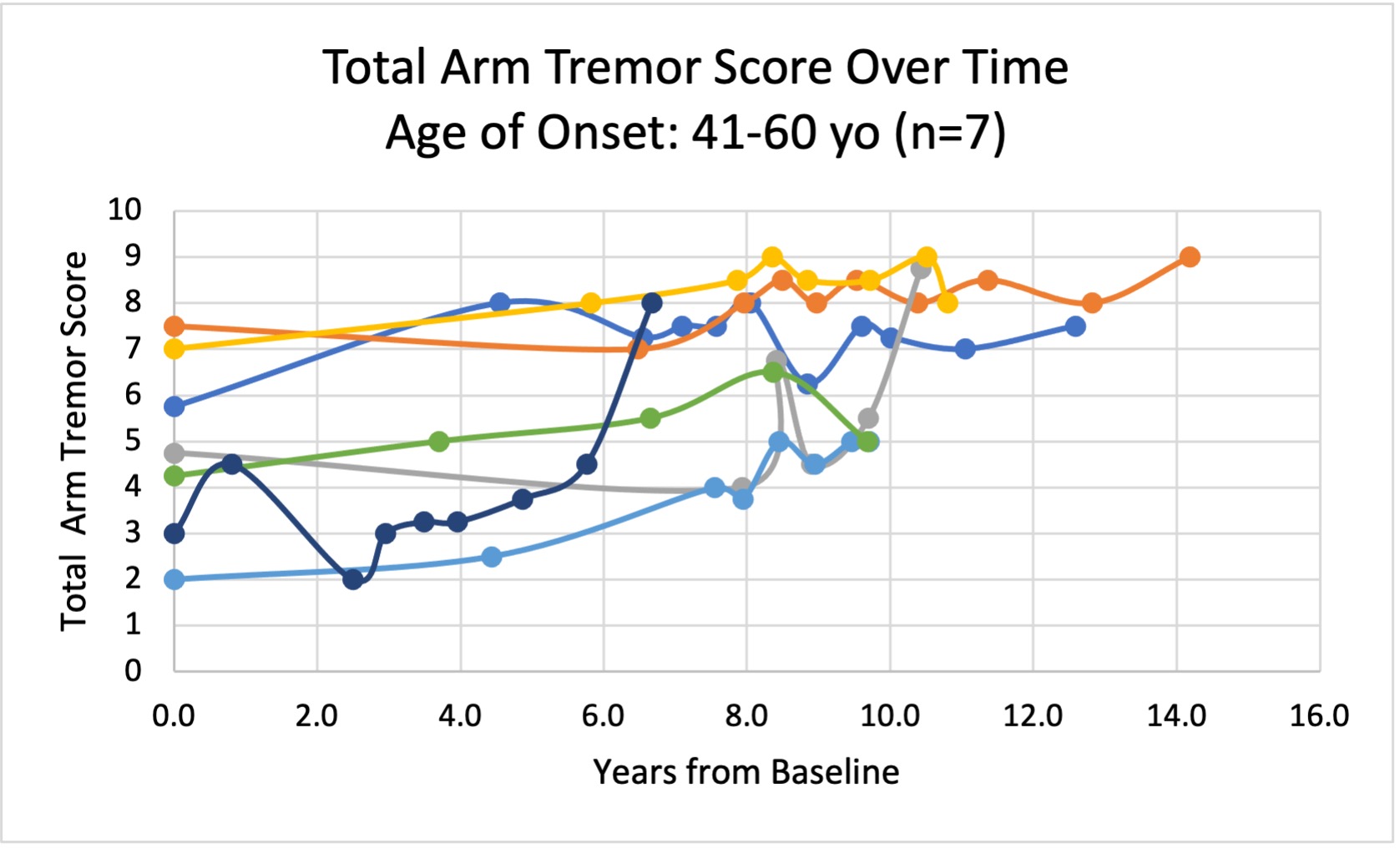

Supplement: Supplementary file 3 [file Image_3.JPEG]

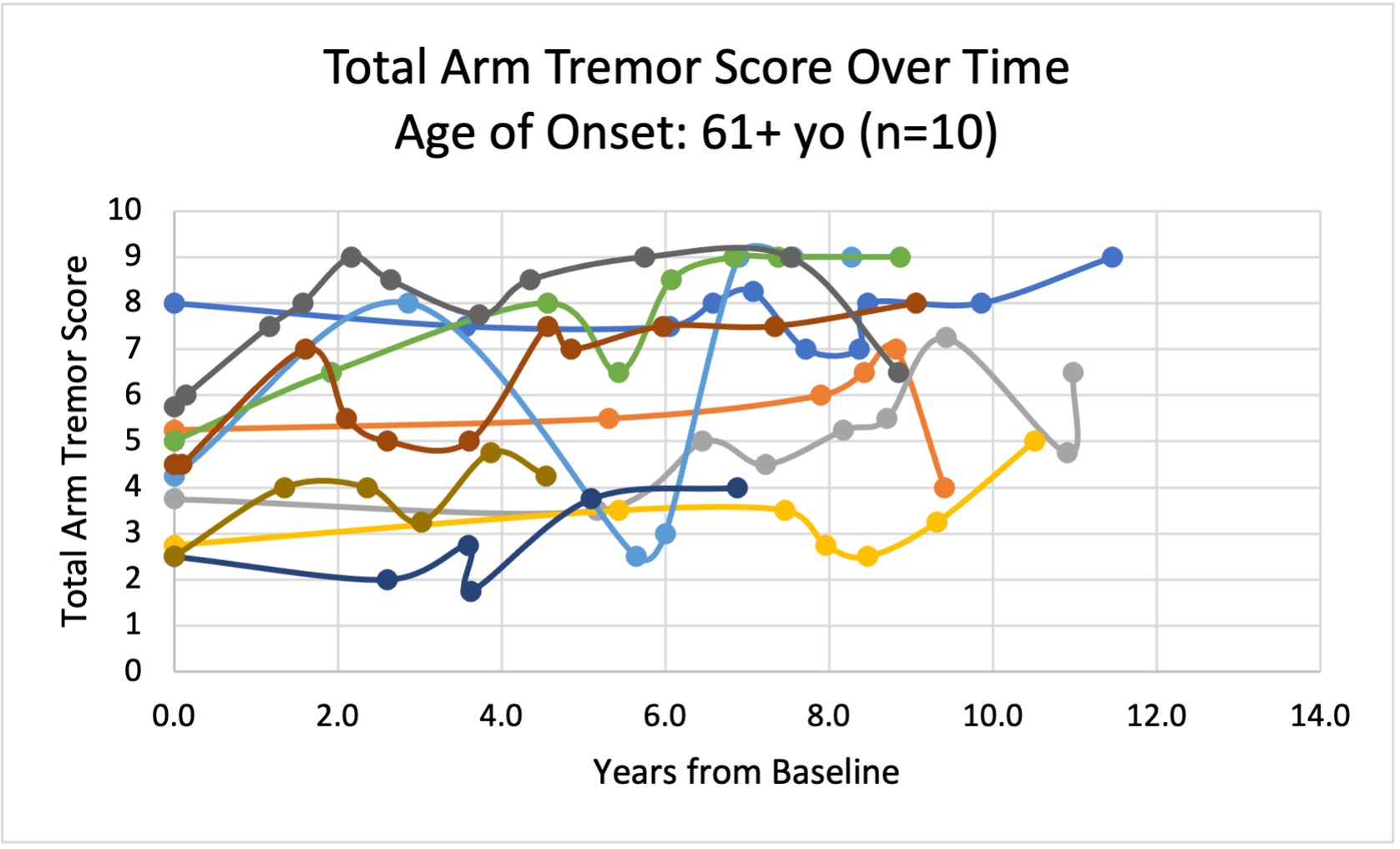

Supplement: Supplementary file 4 [file Image_4.JPEG]
